# Resin-Based Technology for the Efficient Removal of Benzocaine from Wastewaters

**DOI:** 10.3390/polym18091082

**Published:** 2026-04-29

**Authors:** Nicoleta Mirela Marin, Toma Galaon, Adriana Mariana Borș, Ludmila Motelica, Ovidiu Oprea

**Affiliations:** 1 National Research and Development Institute for Industrial Ecology ECOIND, Street Podu Dambovitei No. 57-73, District 6, 060652 Bucharest, Romania; tomagalaon@yahoo.com; 2 Department of Analytical and Physical Chemistry, University of Bucharest, 4-12 Regina Elisabeta Bd., 030018 Bucharest, Romania; 3 Department of Oxide Materials Science and Engineering, National University of Science and Technology Politehnica Bucharest, 1-7 Gh. Polizu, 060042 Bucharest, Romania; 4 National Institute for R&D for Optoelectronics-Subsidiary, Research Institute for Hydraulics and Pneumatics—INOE 2000-IHP, 040558 Bucharest, Romania; bors.ihp@fluidas.ro; 5 National Centre for Micro- and Nanomaterials, National University of Science and Technology Politehnica Bucharest, 313 Independence Boulevard, 060042 Bucharest, Romania; motelica_ludmila@yahoo.com (L.M.); ovidiu.oprea@upb.ro (O.O.); 6 National Centre for Food Safety, National University of Science and Technology Politehnica Bucharest, 313 Independence Boulevard, 060042 Bucharest, Romania; 7 Academy of Romanian Scientists, 3 Ilfov St., 050045 Bucharest, Romania; 8 Department of Inorganic Chemistry, Physical Chemistry and Electrochemistry, Faculty of Chemical Engineering and Biotechnologies, National University of Science and Technology Politehnica Bucharest, 1-7 Gh. Polizu St., 011061 Bucharest, Romania

**Keywords:** benzocaine, Amberlite XAD-7, experimental parameters, adsorption, FTIR-ATR, TG/DSC

## Abstract

Pharmaceutical residues continue to increasingly contaminate water systems at a global level, and conventional wastewater treatment plants are unable to completely remove these emergent compounds. This study investigates the benzocaine adsorption from aqueous solutions onto Amberlite XAD-7 (X7) resin, with emphasis on quantitative performance metrics and mechanistic understanding. Adsorption occurred rapidly, reaching equilibrium within 60 min, with a maximum adsorption capacity (*Q_e_*) of 140 mg/g and a Langmuir monolayer capacity of 147 mg/g. Experimental parameters strongly influenced X7 performance: resin dosage (0.01–0.05 g) and agitation speed (25–200 rpm) enhanced removal efficiency from 10% to 99.9%, while pH variation (5–9) had a negligible effect, confirming a predominantly hydrophobic, non-ionic adsorption mechanism. Equilibrium data are best described by the Langmuir model (R^2^ = 0.9920, b = 5.2 L/mg, RL = 0.0003), indicating highly favorable monolayer adsorption, while kinetic behavior is described by the pseudo-second-order (PSO) model. FTIR-ATR analysis confirms benzocaine retention through characteristic shifts in aromatic, amine, and ester bands. TG/DSC measurements prove the thermal stability of X7 and the incorporation of benzocaine within the polymeric matrix. Desorption efficiencies ranged from 40% (NaOH) to 97% (HCl-ethanol mixture), demonstrating that X7 was regenerated under the tested conditions with a single cycle. Overall, X7 exhibits high capacity, robustness, and recyclability, highlighting its strong potential for efficient benzocaine removal from contaminated wastewater.

## 1. Introduction

The continuous release of pharmaceutical compounds into aquatic environments has emerged as a critical global concern due to their persistence, bioactivity, and potential ecological and human health impacts [1,2,3,4]. Pharmaceuticals such as antibiotics [5,6], analgesics [7,8], antiepileptics [9,10], and antiarrhythmics [11,12] are frequently detected in wastewater effluents, surface waters, and even drinking water sources, reflecting their incomplete removal in conventional treatment systems [13,14]. Their chronic presence, even at trace concentrations, can contribute to antimicrobial resistance, endocrine disruption, and long-term ecological imbalance. Consequently, the development of efficient, cost-effective, and regenerable adsorbent materials for drug removal has become a major research priority [15,16,17,18,19,20].

Among the various adsorption materials investigated, polymeric resins, particularly the Amberlite XAD series, have gained significant attention due to their tunable polarity, high surface area, chemical stability, and reusability [21,22,23]. X7, an acrylic ester resin with moderate polarity, has demonstrated strong affinity for a wide range of organic pollutants, including pharmaceuticals, pesticides, and industrial chemicals. Its physicochemical characteristics—such as pore structure, surface heterogeneity, and compatibility with aqueous systems—make it especially suitable for removing moderately polar compounds that are not efficiently retained by nonpolar resins [24,25,26]. Recent studies have highlighted the effectiveness of X7 and its modified forms in removing pharmaceuticals from water matrices [27,28,29,30,31]. For example, the adsorption of common drugs such as carbamazepine, naproxen, ketoprofen, and trimethoprim onto X7 has been shown to follow Langmuir or Freundlich isotherms, with capacities reaching up to 97 mg/g depending on pH and the molecular properties of the target compound. Kinetic investigations further indicate that adsorption often follows pseudo-second-order behavior, suggesting chemisorption or strong surface interactions, as demonstrated in studies examining the removal of carbamazepine, naproxen, and trimethoprim [27]. Additionally, the resin has been successfully applied for the removal of antiarrhythmic drugs such as procainamide and lidocaine, confirming its versatility across different pharmaceutical classes [28]. Beyond pharmaceuticals, X7 has also been used for the adsorption of metal ions when it is impregnated with specific extractants, demonstrating its adaptability for multifunctional water treatment applications [28,31,32].

Despite these promising results, the adsorption performance of X7 can differ significantly depending on solution chemistry, competitive solutes, and the physicochemical properties of target drugs. Factors such as pH, ionic strength, molecular charge, and hydrophobicity influence adsorption mechanisms and capacities, necessitating systematic evaluation for each class of contaminants. Moreover, the presence of multiple pharmaceuticals in real wastewater streams can lead to competitive adsorption effects, altering removal efficiencies and requiring deeper mechanistic understanding. Given the increasing complexity of pharmaceutical pollution and the need for robust treatment technologies, further investigation into the adsorption behavior, regeneration potential, and practical applicability of X7 is essential.

In this study, X7 was selected due to its unique mixture of polarity, surface chemistry, and retention performance, making it particularly useful for the removal of slightly polar pharmaceutical compounds, such as benzocaine. X7 is an acrylic polymer that exhibits a high surface area and porosity, with moderate hydrophilic behavior and intermediate polarity. This polarity pattern sets it aside from many commonly used adsorbents and fits well with the physical–chemical properties of benzocaine, which is largely neutral and hydrophobic after pH 2.5.

To the best of the authors’ knowledge, this is the first study to investigate benzocaine removal using X7 through an integrated approach that combines operational optimization, adsorption and kinetic equilibrium, and spectroscopic and thermal characterization, thereby contributing to the development and optimization of resin-based adsorption systems for wastewater treatment applications.

## 2. Materials and Methods

### 2.1. Materials

X7 resin purchased from Sigma-Aldrich, St. Louis, MO, USA has a moderately hydrophilic behavior, with an intermediate value of polarity; it is an acrylic polymer that possesses a developed surface and porosity. Polymeric resins have many advantages because they can be used in the entire pH range and do not contain interfering ionizable groups [33].

**Figure 1:**
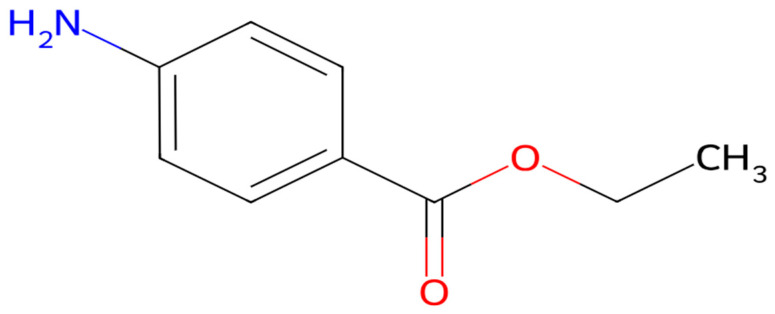
Two-dimensional structure of benzocaine.

Benzocaine C_9_H_11_NO_2_ (Sigma Aldrich)—an ethyl ester of p-aminobenzoate (ethyl p-aminobenzoate or ethyl 4-aminobenzoate)—is a fast-acting local ester-type anesthetic that acts on nerve impulses. It is a benzoate ester with the component 4-aminobenzoic acid: an ethanol and a substituted aniline. Benzocaine (Figure 1) is characterized by steric and aromatic amine-type functional groups, with a melting point around 90 °C and a molecular weight of 165.19 g/mol. It is insoluble in water (pKa 2.5), especially in neutral or basic solutions, but very soluble in alcohol, ether, and chloroform, and can form a charged salt under acidic conditions [34].

### 2.2. Methods

The thermal analysis (TG-DSC) for the resins and benzocaine was performed with a Netzsch STA 449C Jupiter apparatus (NETZSCH-Gerätebau GmbH, Selb, Germany). The samples were placed in an open crucible made of alumina and heated at 10 K·min^−1^ from room temperature to 900 °C, under the flow of 50 mL min^−1^ dried air. An empty alumina crucible was used as reference.

FTIR analyses of X7, solid benzocaine, and X7 loaded with benzocaine were performed using a Nicolet iS50R spectrometer (Thermo Fisher Scientific Inc, Madison, WI USA) at room temperature using the Attenuated Total Reflectance (ATR) module. This method involves the use of crystal, on which the test sample is applied. In total, 32 scans of the samples between 4000 and 400 cm^−1^ were performed, at a resolution of 4 cm^−1^.

The quantitative determination of benzocaine was performed by UV–Vis spectrometry using the calibration curve obtained in a concentration range that was varied from 10 to 30 mg/L.

All adsorption–desorption experiments were carried out in a controlled batch system, where a fixed mass of X7 resin was brought into contact with benzocaine solutions to evaluate the influence of operational parameters (contact time, sorbent dosage, agitation speed, pH, and initial concentration). Each experimental condition was tested in duplicate to ensure reproducibility, and the mean benzocaine concentrations were used to calculate the adsorption capacity of the X7 resin. All measurements exhibited standard deviations below 3%.

### 2.3. Procedure for Study the Kinetic of Adsorption Process

To investigate the kinetics of benzocaine adsorption onto the X7 resin, fixed amounts of resin (~0.05 g) were stirred with 0.01 L of 500 mg/L benzocaine solution. The resulting mixtures were maintained under stirring from 0 to 90 min. In order to study the influence of contact time on the adsorption process, the equilibrium concentration of benzocaine in solution was determined by UV–Vis method. The time required to reach adsorption equilibrium was defined as the minimum contact time at which the benzocaine concentration remained unchanged.

The amount of benzocaine adsorbed at time *t*, reported at each mass of resin, was calculated using Equation (1) [28].

(1) $$Q_{t}=\frac{\left(C_{i}-C_{t}\right)V}{m}$$

where *Q_t_* (mg/g) represents the amount of benzocaine adsorbed per gram of X7 resin at different interval times. *C_i_* (mg/L) is the initial benzocaine concentration in solution, while *C_t_* (mg/L) is the benzocaine concentration at time *t*. Also, *V*(L) is the volume of the solution used for each sample, and m(g) is the mass of resin maintained in contact with the benzocaine solution.

### 2.4. Procedure for Dosage Mass Influence

A batch adsorption experiment was conducted to evaluate the influence of adsorbent dose on solute removal. For each test, 10 mL of 500 mg/L solution was brought into contact with varying sorbent masses (0.01–0.05 g) in Erlenmeyer flasks. The suspensions were agitated for 60 min at T = 25 ± 2 °C, after which the solid and liquid phases were separated by filtration. The final solute concentration in the supernatant was measured to determine adsorption capacity *Q_e_* (mg/g) or removal efficiency *R* (%) as a function of adsorbent dosage.

The adsorption capacity *Q_e_* (mg/g) and removal efficiency *R* (%) were calculated based on Equations (2) and (3) [28].

(2) $$Q_{e\;}=\frac{\left(C_{i}-C_{e}\right)\;V}{m}$$

(3) $$R\;\left(\%\right)=\frac{C_{i}-C_{e}}{C_{i}}\times 100$$

where *C_e_* (mg/L) represents the benzocaine concentration in solution at equilibrium at the end of the stirring time.

### 2.5. Procedure for Influence of Stirring Speed

A batch adsorption experiment was performed to investigate the influence of stirring speed on solute removal. In each test, 0.05 g of X7 was added to 10 mL of a 500 mg L^−1^ solution in an Erlenmeyer flask, and the suspension was agitated for 60 min at T = 25 ± 2 °C using different stirring speeds (25, 50, 100, 150, and 200 rpm). At the end of the contact time, the solid and liquid phases were separated by filtration, and the final solute concentration in the supernatant was determined to evaluate the effect of stirring speed on benzocaine removal efficiency.

### 2.6. Procedure for pH Influence

A batch adsorption experiment was carried out to evaluate the influence of pH on benzocaine removal. Solutions with initial concentrations between 100 and 700 mg L^−1^ were prepared, and their pH values were adjusted to 5, 7, or 9 using 1 M HCl and NaOH. For each condition, 0.05 g of X7 was added to 10 mL of solution in an Erlenmeyer flask and agitated at 150 rpm for 60 min at T = 25 ± 2 °C. After equilibration state was reached, the suspensions were subjected to solid–liquid separation by filtration, and the final solute concentration in the supernatant was measured to assess the effect of pH on removal efficiency.

### 2.7. Procedure for Testing Influence of Initial Concentration

A batch adsorption experiment was conducted to investigate the influence of initial benzocaine concentration on removal efficiency. A series of solutions with initial concentrations of 100, 200, 300, 400, 500, 600, and 700 mg L^−1^ were prepared, and for each condition, 0.05 g of X7 was brought into contact with 10 mL of solution in an Erlenmeyer flask. The suspensions were agitated at 150 rpm for 60 min at T = 25 ± 2 °C, after which solid–liquid separation was performed by filtration. The final solute concentration in the supernatant was determined to evaluate the effect of initial concentration on the adsorption performance.

### 2.8. Procedure for Influence of Desorption Agents

A desorption study was conducted to assess the effectiveness of different desorption agents on the regeneration of the X7 resin loaded with benzocaine. Pre-saturated sorbent samples (0.01 g) were brought into contact with 10 mL of each desorbing solution—1 M NaOH, 1 M HCl, ethanol, and a 1 M HCl–ethanol mixture—and agitated at 150 rpm for 30 min at T = 25 ± 2 °C. Following agitation, the suspensions were subjected to solid–liquid separation by filtration, and the concentration of benzocaine desorbed in the supernatant was quantified to determine the desorption efficiency of each agent.

## 3. Results and Discussion

In the experimental investigations, the influence of key operational parameters on the efficiency of benzocaine retention by the X7 resin was systematically evaluated. Subsequently, the adsorption process was examined in detail through the application of adsorption isotherms and kinetic models.

### 3.1. Influence of Contact Time

**Figure 2:**
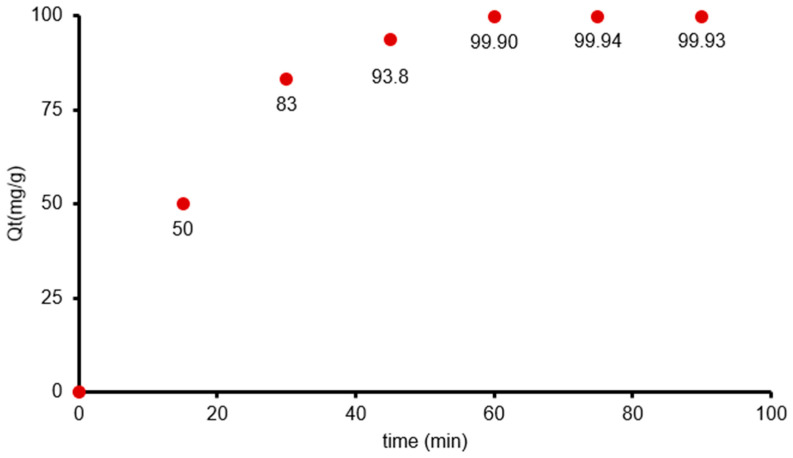
Influence of contact time on benzocaine adsorption onto X7 resin mass. All reported values correspond to the mean of two duplicate experiments, each exhibiting a standard deviation below 3%.

The effect of contact time on the adsorption of benzocaine onto X7 was evaluated over a 90-min period, and the results demonstrate a characteristic adsorption profile consisting of an initial rapid uptake followed by a gradual approach to equilibrium (Figure 2). At the onset of the experiment at 0 min, no benzocaine adsorption was observed, as expected. However, within the first 15 min, the concentration of benzocaine in solution decreased from 500 mg/L to 400 mg/L, corresponding to a significant increase in adsorption capacity (*Q_t_* = 50 mg/g). This rapid initial uptake can be attributed to the abundance of available active sites on the resin surface and the strong concentration gradient driving mass transfer from the bulk solution to the adsorbent.

Between 15 and 45 min, the adsorption rate remained substantial but progressively slowed. After 30 min, *C_e_* had decreased to 250 mg/L, and *Q_e_* increased to 83 mg/g. After 45 min, *C_e_* further declined to 125 mg/L, with *Q_t_* reaching 93.8 mg/g.

A near-equilibrium state was achieved at approximately 60 min, where *C_e_* decreased to 0.5 mg/L and *Q_e_* reached 99.90 mg/g. Extending the contact time to 75 and 90 min resulted in only marginal changes in *C_e_* (0.3–0.35 mg/L) and *Q_t_* (99.93–99.94 mg/g), indicating that the adsorption process had effectively reached equilibrium. The minimal variation in adsorption capacity beyond 60 min suggests that the majority of active sites were saturated and that the driving force for further adsorption had diminished.

Overall, the data indicate that X7 exhibits a high affinity for benzocaine, achieving nearly complete removal within 60 min. The adsorption kinetics are characterized by a rapid initial phase dominated by external mass transfer, followed by a slower intraparticle diffusion phase, leading to equilibrium. These findings imply that a contact time of 60 min is sufficient to achieve maximum adsorption efficiency under the experimental conditions and that longer contact times do not yield significant additional uptake.

### 3.2. Influence of X7 Dosage

**Figure 3:**
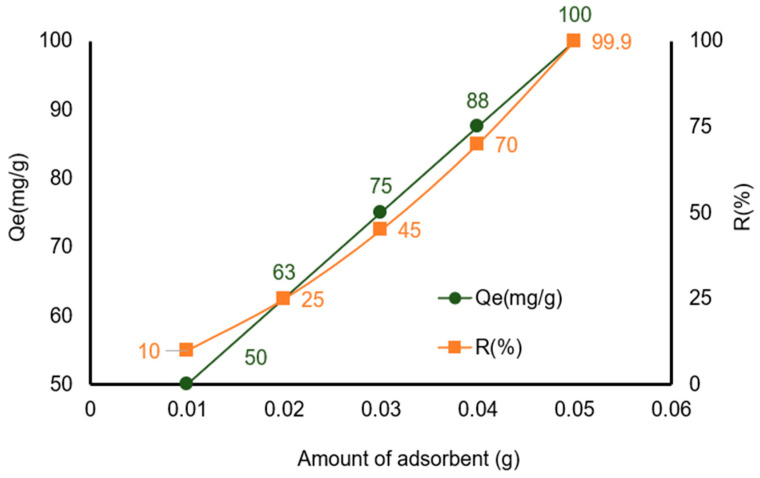
Influence of amount adsorbent for benzocaine adsorption onto X7 resin mass. All reported values correspond to the mean of two duplicate experiments, each exhibiting a standard deviation below 3%.

The results clearly show that increasing the X7 dosage significantly enhances the removal efficiency of the benzocaine (Figure 3). Removal efficiency increases with dosage, and it was observed that, at 0.01 g, removal is only 10%, and at 0.05 g, removal reaches 99.9%. This trend is expected because adding more X7 increases the number of available active sites, allowing more benzocaine molecules to be captured. Equilibrium concentration decreases from 450 mg/L to 0.35 mg/L as dosage increases. This indicates a strong adsorption affinity and efficient benzocaine uptake at higher dosages.

Thus, increasing the X7 dosage improves removal efficiency due to the greater availability of adsorption sites. However, the adsorption capacity per gram does not increase proportionally at higher dosages because the benzocaine concentration becomes insufficient to fully utilize all active sites.

### 3.3. Effect of Agitation Speed on Adsorption Experiment

**Figure 4:**
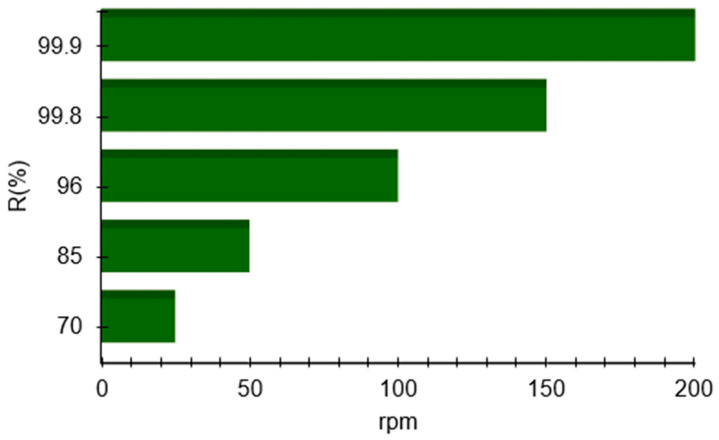
Influence of agitation speed for benzocaine adsorption on X7 resin. All reported values correspond to the mean of two duplicate experiments, each exhibiting a standard deviation below 3%.

The experimental results demonstrate a clear dependence of adsorption performance on agitation speed. As shown in Figure 4, increasing the agitation rate from 25 to 200 rpm resulted in a substantial decrease in *C_e_* from 150 mg/L to 0.5 mg/L. Correspondingly, both *Q_e_* and *R* obviously increased, reaching values as high as 100 mg/g and 99.9%, respectively, at the highest agitation speed. At lower agitation speeds (25–50 rpm), the system exhibited relatively modest removal efficiencies (70–85%). This behavior is characteristic of mass-transfer-limited adsorption, where insufficient mixing leads to the formation of a thicker hydrodynamic boundary layer around the X7 particles. Under such conditions, the diffusion of solute molecules from the bulk solution to the X7 surface is inhibited, resulting in lower adsorption rates and capacities.

As agitation speed increased to 100 rpm, the removal efficiency increased sharply to 96%, indicating a significant enhancement in external mass transfer. The reduction in boundary layer thickness and improved dispersion of X7 particles facilitated more-effective contact between solute molecules and active adsorption sites. At agitation speeds of 150 and 200 rpm, the system approached near-complete solute removal (99.8–99.9%). These results suggest that, at high mixing intensities, external mass-transfer resistance becomes negligible, allowing the adsorption process to proceed at or near its intrinsic kinetic and adsorption limits. The X7’s full capacity is effectively utilized under these conditions. Therefore, the data obtained indicate that agitation speed plays a critical role in controlling the rate and extent of adsorption. The observed trends are consistent with classical adsorption theory, where external film diffusion is often the dominant resistance at early stages of adsorption [35]. By increasing agitation, this resistance is minimized, enabling the X7 to achieve its maximum potential performance.

### 3.4. Influence of pH Solution

The central objective of this study was to evaluate whether pH influences the adsorption of benzocaine onto X7. The pH interval evaluated in this study (5–9) was selected to reflect environmentally relevant conditions for wastewater treatment and does not reflect any material-related constraints, as the resin is stable and functional across the full 0–14 pH range.

Adsorption onto this acrylic ester resin is primarily driven by hydrophobic and specific non-electrostatic interactions.

X7 exhibits a zero-charge point (pHpzc) between 6.2 and 6.8, meaning that its surface is slightly positively charged at pH values below pHpzc and slightly negatively charged above it [27,36]. Because the experimental pH interval (5–9) spans both sides of the pHpzc, the minimal variation observed in adsorption capacity indicates that electrostatic contributions are negligible. This behavior aligns with the predominantly hydrophobic and nonionic adsorption mechanism previously reported for this class of resins.

Benzocaine contains a para-amino group whose conjugate acid has a pKa of approximately 2.5–3.0. Thus, at pH < pKa, benzocaine is mainly present as the protonated cation (–NH_3_^+^), whereas at pH > pKa it exists predominantly in its neutral form (-NH_2_), which is more hydrophobic and less soluble in water. Since adsorption onto XAD-7 is governed largely by hydrophobic interactions, the neutral species of benzocaine exhibits a markedly stronger affinity for the resin. The fact that adsorption remains high across the entire pH range tested confirms that the neutral form dominates under these conditions and that hydrophobic interactions control the retention process.

Although X7 is formally non-ionic, residual functional groups on the acrylic matrix impart a measurable point of zero charge (pHPZC), typically reported in the near-neutral range (≈6–7) for acrylic adsorbents. When pH < pH_PZC_, the resin surface tends to be slightly positively charged. Also, when pH > pH_PZC_, the resin surface becomes slightly negatively charged and can influence adsorption when benzocaine is in its protonated form.

**Figure 5:**
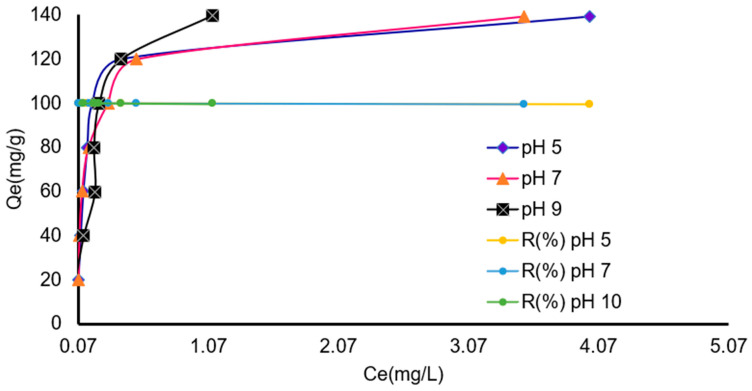
Influence of initial concentration and pH on the benzocaine removal on the X7 resin. All reported values correspond to the mean of two duplicate experiments, each exhibiting a standard deviation below 3%.

The data obtained are presented in Figure 5 and reveal that at pH 5, removal efficiencies ranged from 99.4 to 100.0%, with *Q_e_* values from ≈20 to ≈139 mg/g. At pH 7, removal efficiencies remained similarly high (99.5–100.0%), with nearly identical *Q_e_* values. At pH 9, removal efficiencies were slightly higher at the upper concentration range (up to 99.8–100.0%), with *Q_e_* values marginally exceeding those at pH 5 and 7. The minimal differences observed among pH 5, 7, and 9 indicate that pH has no significant influence on benzocaine adsorption within this range. Several factors support this conclusion: X7 is a non-ionic polymeric resin, meaning its surface chemistry is largely unaffected by pH changes. Benzocaine remains predominantly in a neutral molecular form across pH 5–9, reducing the likelihood of electrostatic interactions dominating the adsorption process. The consistently high removal efficiencies suggest that hydrophobic interactions, Van der Waals forces, and π–π interactions between benzocaine and the polymeric matrix are the primary adsorption mechanisms.

The resin exhibited exceptionally high adsorption performance under all conditions, with removal efficiencies exceeding 99% and adsorption capacities reaching approximately 140 mg/g. The negligible variation in *Q_e_* and *R* (%) across the tested pH values demonstrates that the adsorption process is largely independent of pH within this range. This behavior is consistent with a mechanism dominated by hydrophobic and non-ionic interactions rather than electrostatic effects. Consequently, X7 can be considered a robust and highly effective adsorbent for benzocaine removal from aqueous media without the need for stringent pH control.

### 3.5. Influence of Initial Concentration at Different pH Values

Across all three pH values (5, 7, and 9), the experimental results show a consistent pattern: *Q_e_* increases proportionally with *C_i_*, indicating that higher initial concentrations provide a stronger driving force for mass transfer. Removal efficiency (*R*(%)) remains extremely high (≈99.8–100%) at all pH values and concentrations. *C_e_* values remain very low, confirming near-complete adsorption even at the highest *C_i_* (700 mg/L). This suggests that the adsorbent possesses very high affinity and large available capacity under all tested pH conditions (Figure 5).

At pH 5, *Q_e_* increases linearly from 19.99 mg/g (*C_i_* = 100 mg/L) to 139.70 mg/g (*C_i_* = 700 mg/L). Also, *R*% remains at 99.8–100%, showing that, even at low pH, the adsorbent surface remains highly active. Competition with H^+^ ions does not significantly inhibit the adsorption process. This is notable because many adsorbents show reduced performance at low pH due to the protonation of surface functional groups.

At pH 7, *Q_e_* values are nearly identical to those at pH 5, and adsorption varied from 19.99 mg/g at *C_i_* = 100 mg/L to 139.68 mg/g at *C_i_* = 700 mg/L. This suggests that neutral pH does not alter the adsorption mechanism significantly. The adsorbent surface may be near its point of zero charge (PZC), minimizing repulsion and allowing for efficient uptake [27,36]. *R*% remains at 99.8–100%, confirming excellent adsorption capacity in neutral conditions and the minimal influence of hydroxyl or proton competition.

At pH 9, *Q_e_* ranged from 20.0 mg/g at *C_i_* = 100 mg/L to 139.7 mg/g at *C_i_* = 700 mg/L. This indicates that adsorption is not affected by an increase in the concentration of OH^−^. The concentration range analyzed in this study (100–700 mg L^−1^) reflects the typical conditions of high-concentration industrial wastewater streams associated with pharmaceutical manufacturing. Under these conditions, X7 demonstrated exceptionally high adsorption performance, achieving 99.9% removal at the lowest tested concentration (100 mg/L), which allowed us to extend the concentration range studied up to 700 mg/L, where the saturation plateau of the tested resin was reached. This result suggests that X7 has a high affinity for benzocaine, underscoring its potential suitability for treating high-load industrial effluents before subsequent dilution or discharge. Concentrations of pharmaceutical products in industrial effluents can reach high levels, particularly in manufacturing or formulation operations. For example, one literature study reported concentrations of active pharmaceutical ingredients ranging from tens to hundreds of mg/L in untreated pharmaceutical wastewater streams [37,38]. Even though specific reports on benzocaine are limited, these studies confirm that scenarios with high loads of this magnitude are realistic for industrial discharges.

We also note that all experiments were conducted at a single temperature (25 ± 2 °C). Consequently, thermodynamic parameters such as ΔG°, ΔH°, and ΔS° could not be calculated, as their determination requires equilibrium data collected at multiple temperatures. Thus, this study focuses on equilibrium and kinetic modeling at a fixed temperature, and multitemperature thermodynamic analysis is not the subject of this paper.

Furthermore, although a detailed economic assessment was not the focus of this study, the high adsorption capacity and minimal pH dependence suggest that X7 could be a cost-effective option for the removal of benzocaine under real-world wastewater treatment conditions.

#### 3.5.1. Studies of Isotherm Models

The adsorption behavior of the studied system was evaluated using Langmuir (Equations (4) and (5)), Freundlich (Equation (6)), Temkin–Pyzhev (Equations (7) and (8)), and Dubinin–Radushkevich (D–R) (Equations (9)–(11)) isotherm models [35,36]. The obtained parameters (Table 1) provide insight into the adsorption mechanism, surface characteristics, and nature of the adsorbent–adsorbate interactions.

(4) $$\frac{C_{e}}{Q_{e}}=\frac{1}{bQ_{0}}+\frac{C_{e}}{Q_{0}}$$

(5) $$R_{L}=\frac{1}{1+bC_{0}}$$

(6) $$\ln Q_{e\;}=\ln K_{f}\;+\frac{1}{n}{\mathrm{lnC}}_{e}$$

(7) $$Q_{e}=\frac{\mathrm{RT}}{b_{T}}\ln\;(AC_{e})$$

and can be linearized as follows:

(8) $$Q_{e}\;=\;\mathrm{BlnA}\;+\;\mathrm{Bln}C_{e}$$

(9) $$\ln Q_{e}=\ln q_{m}-\beta \varepsilon^{2}$$

(10) $$\varepsilon =\mathrm{RTln}(1+\frac{1}{C_{e}})$$

(11) $$the\;E=\frac{1}{\sqrt{2\beta }}$$

where *Q*_0_ (mg/g) represents the adsorption capacity of the X7 resin. *b* (L/mg) is the Langmuir constant, which reflects the affinity between the X7 and the benzocaine. *R_L_* is the separation factor (a dimensionless constant) used to evaluate the favorability of the sorption process. *R*^2^ is the correlation coefficient, indicating the goodness of fit of the applied isotherm model. *K_f_* (mg/g) is the Freundlich constant associated with the sorption capacity of the resin. n is the Freundlich exponent, which describes the intensity of the sorption process. *b_T_* (J/mol) is the Temkin constant, related to the heat of sorption. *q_m_* (mg/g) represents the theoretical monolayer sorption capacity calculated using the Dubinin–Radushkevich equation. *E* (kJ/mol) is the mean sorption energy.

X7 is a macroporous acrylic resin with a heterogeneous pore-size distribution and a complex surface; this is one reason it does not strictly adhere to the theoretical assumptions of the Langmuir model. However, the Langmuir isotherm was used as an analytical model that provides the best fit to the experimental data (*R*^2^ = 0.9920)—not as evidence of a perfectly homogeneous monolayer adsorption mechanism. At *Q*_0_ = 147 mg/g, this relatively high value of monolayer capacity suggests strong affinity of the adsorbent for the adsorbate and a well-developed macroporous surface. The low RL value (0.0003) and Langmuir constant (b = 5.2 L/mg) reflect the strong affinity between benzocaine and X7 under the experimental tested conditions, as well as more-than-ideal surface uniformity.

The Freundlich model describes adsorption on heterogeneous surfaces and multilayer adsorption. However, the model shows a poor fit to the data (*R*^2^ = 0.5424), suggesting that surface heterogeneity is not the dominant mechanism. *K_f_* = 116 mg/g indicates relatively high adsorption capacity, consistent with Langmuir results. Also, *n* = 2.75, indicating favorable adsorption (*n* > 1). This value indicates moderate surface heterogeneity. Moreover, *1*/*n* = 0.36 (a value between 0 and 1 indicates favorable adsorption), but the relatively low *R*^2^ suggests that Freundlich does not adequately describe the adsorption behavior. Thus, while the Freundlich constants indicate favorable adsorption, the poor model fit suggests that multilayer adsorption or strong heterogeneity are not the primary mechanisms.

The Temkin–Pyzhev model accounts for adsorbate–adsorbent interactions and assumes that the heat of adsorption decreases linearly with coverage. The moderate correlation coefficient (*R*^2^ = 0.741) indicates partial applicability. A relatively high Temkin constant of *A* = 88.4 L/mg suggests strong binding interactions. Furthermore, *b_T_* = 112 J/mol reflects the heat of adsorption. Values below 8 kJ/mol typically indicate physisorption, but Temkin’s *b_T_* is not directly comparable to D–R energy values. Still, the moderate magnitude suggests weak to moderate interactions. *B* = 26.7 indicates the influence of adsorbate–adsorbent interactions on adsorption behavior. The Temkin model suggests that adsorption energy decreases gradually with surface coverage, consistent with real systems, but it does not outperform Langmuir in describing the data.

The D–R model provides insight into the adsorption mechanism and distinguishes between physical and chemical adsorption. The model shows a reasonably good fit (*R*^2^ = 0.8475); it is better than Freundlich and Temkin but still inferior to Langmuir. The higher value of theoretical capacity obtained for the D–R model (*q_m_* = 183 mg/g) compared to the Langmuir monolayer capacity (*Q*_0_ = 147 mg/g) has been recognized as an indication of surface heterogeneity and the presence of multiple adsorption sites within the resin’s porous structure. Additionally, *β* = 3.98 × 10^−8^ mol^2^/kJ^2^ represents a very low *β* value, indicating low adsorption energy. Here, *E* = 3.54 kJ/mol, and since *E* < 8 kJ/mol, the adsorption process is classified as physisorption, dominated by weak Van der Waals forces. The D–R results confirm that the adsorption mechanism is physical rather than chemical, consistent with the low *R_L_* value and moderate Temkin heat of adsorption.

#### 3.5.2. Kinetics of Adsorption Process

The adsorption kinetics of benzocaine on X7 were evaluated using pseudo-first-order (PFO) (Equation (12)) and pseudo-second-order (PSO) (Equation (13)) models to elucidate the mechanisms that determine the rate of the process (Table 2) [26].

(12) $$log(q_{e}-q_{t})=log\;q_{e}-\frac{k_{1}}{2.303}t$$

(13) $$\frac{t}{q_{t}}=\frac{1}{k_{2}q_{e}^{2}}+\frac{t}{q_{e}}$$

The linearized PFO model yielded a correlation coefficient of *R*^2^ = 0.955, indicating that, although the model reflects the general trend of benzocaine adsorption, it does not accurately reproduce the whole kinetic profile. The calculated equilibrium adsorption capacity (*q*_*e*,*calc*_ = 98.7 mg/g) deviated from the experimental value (≈100 mg/g), suggesting that the PFO model does not adequately describe the rate-limiting step. This disagreement is consistent with cases where adsorption is not governed strictly by diffusion processes at the external surfaces.

In contrast, the PSO model fits the experimental data exceptionally well, with a correlation coefficient of *R*^2^ = 0.9990. The calculated equilibrium capacity (*q*_*e*,*calc*_ = 100.4 mg/g) agrees very well with the experimental value *Q_t_*, confirming the robustness of the model. The PSO model indicates that the adsorption rate is primarily controlled by surface interactions involving electron sharing or transfer between the benzocaine molecules and the X7 polymer matrix. This behavior is characteristic of systems in which specific interactions or other strong interactions dominate the adsorption mechanism.

The kinetic results are in full agreement with the isotherm analysis, with the Langmuir model showing the highest correlation (*R*^2^ = 0.992), which confirms monolayer adsorption on a relatively homogeneous surface. The agreement between the PSO kinetic model and the Langmuir isotherm further supports the conclusion that benzocaine adsorption on X7 occurs through strong, localized interactions rather than through multilayer processes or on heterogeneous surfaces.

Although the adsorption kinetics followed a pseudo-second-order (PSO) model, this does not imply chemisorption. The PSO model often describes systems in which the adsorption rate is determined by the availability of active sites or by strong, non-covalent interactions, rather than by chemical bonds. The average Dubinin–Radushkevich sorption energy (E = 3.54 kJ/mol) clearly falls within the physisorption range, indicating that the retention of benzocaine on X7 is dominated by hydrophobic and Van der Waals interactions. Therefore, the PSO kinetic fit and low D–R energy are not contradictory; on the contrary, together they describe a process characterized by rapid adsorption and strong physical interactions, without the formation of chemical bonds.

To conclude, the kinetic evaluation demonstrates that the pseudo-second-order model provides the most accurate description of benzocaine adsorption on X7, reflecting a mechanism dominated by strong adsorbent–adsorbate interactions and a rapid establishment of equilibrium. The good agreement between the experimental and calculated parameters highlights the great affinity and efficiency of X7 for removing benzocaine under the conditions studied.

### 3.6. Desorption Studies

**Figure 6:**
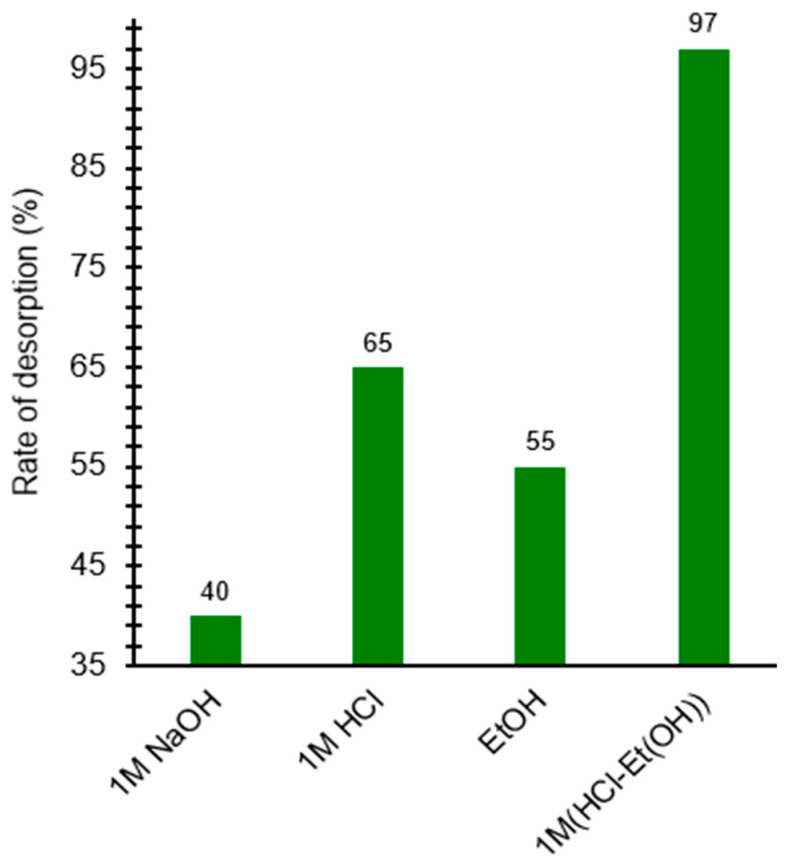
Influence of desorption agents for benzocaine desorption from X7 mass. All reported values correspond to the mean of two duplicate experiments, each exhibiting a standard deviation below 3%.

The desorption behavior of benzocaine from X7 reflects the interplay between the physicochemical properties of the analyte and the characteristics of the polymeric adsorbent. X7 is a moderately polar, non-ionic acrylic resin whose retention mechanism is dominated by hydrophobic and dispersive interactions within its porous structure. Benzocaine, a weakly basic aromatic ester, can exist in either a neutral or protonated form depending on the pH of the desorbing medium, and this ionization state strongly influences its affinity for the resin. The results regarding influence of desorption agents for benzocaine desorption from X7 mass are presented in Figure 6.

Exposure to strongly alkaline conditions maintains benzocaine predominantly in its neutral, uncharged form. Under these conditions, the molecule retains significant hydrophobic character, which favors continued interaction with the non-ionic acrylic matrix. Consequently, 1 M NaOH provides only limited desorption efficiency (40%), indicating that pH-induced deprotonation does not sufficiently weaken the hydrophobic interactions responsible for adsorption.

In contrast, treatment with 1 M HCl leads to extensive protonation of the amino group, converting benzocaine into a more hydrophilic cationic species. Protonation reduces its affinity for the hydrophobic resin and increases its solubility in the aqueous phase. This shift in ionization state enhances desorption to 65%. However, the aqueous acidic medium alone is insufficient to fully disrupt the hydrophobic interactions within the resin pores, leaving a substantial fraction of benzocaine still retained.

Ethanol, a moderately polar organic solvent, promotes desorption primarily by altering the solvent environment rather than the ionization state of benzocaine. Ethanol can penetrate the resin pores and effectively solvate the neutral benzocaine molecules, thereby competing with the hydrophobic surface of X7. This results in a desorption efficiency of 55%, comparable to that achieved with 1 M HCl but driven by solvent polarity rather than acid–base chemistry.

The combination of 1 M HCl with ethanol yields a markedly synergistic effect, achieving near-quantitative desorption (97%). In this mixed-solvent system, benzocaine is both protonated—reducing its hydrophobic affinity—and simultaneously exposed to an organic modifier that enhances solvation and disrupts resin–analyte interactions. The dual action of ionization control and organic solvent penetration effectively overcomes the hydrophobic retention forces within X7, enabling almost complete elution.

To conclude, the desorption data demonstrate that neither pH adjustment nor organic solvent alone is sufficient to fully reverse benzocaine adsorption on X7. Instead, maximal desorption requires a combined mechanism in which protonation decreases the analyte’s hydrophobicity while ethanol reduces the polarity of the desorbing phase and enhances solvation within the resin matrix. This synergistic effect explains the superior performance of the HCl–ethanol mixture and highlights the importance of simultaneously modulating both analyte ionization and solvent polarity when designing efficient desorption protocols for moderately hydrophobic organic compounds on polymeric adsorbents.

### 3.7. The Competitive Performance of X7 Resin in Benzocaine Adsorption: Practical Relevance Compared to Other Adsorbent Materials

Thus, the adsorption of benzocaine on X7 demonstrated in this study (*Q_e_* ≈ 140 mg/g; *Q*_0_ 147 mg/g) confirms the resin’s high affinity for neutral and hydrophobic molecules, surpassing the performance reported for other micropollutants on the same adsorbent. Although advanced materials (MOFs, β-CD polymers) achieve higher capacities, XAD-7 offers fast kinetics, operational stability, and excellent regenerability, which gives it practical relevance in the treatment of contaminated water. The results presented in Table 3 demonstrate the competitiveness of a commercial adsorbent compared to much more sophisticated systems [27,36,39,40,41,42].

### 3.8. FTIR-ATR Studies

**Figure 7:**
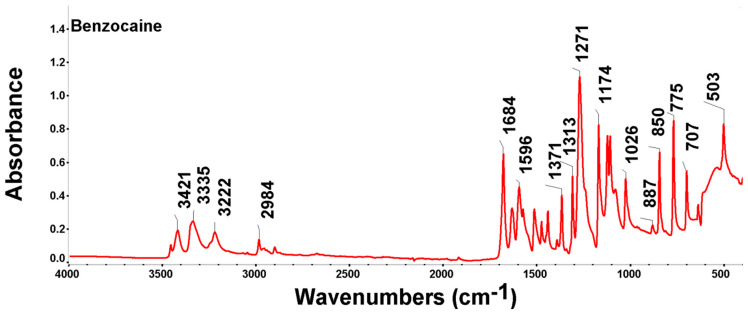
FTIR-ATR spectra of benzocaine.

The FTIR spectrum of benzocaine exhibits characteristic adsorption bands that reflect the presence of the functional groups’ primary amine, ester, and aromatic ring, and the results obtained are presented in Figure 7 and Table 4.

In the range 3417–3220 cm^−1^/3418.35–3220.25 cm^−1^, the vibrational bands typically appear as a well-defined doublet characteristic of primary amines (-NH_2_), indicating an asymmetric/symmetric stretching of these functional groups; the two distinct peaks confirm the presence of a primary amine (not a secondary or tertiary amine).

The appearance of the peak at 3220.25 cm^−1^ is the result of intermolecular hydrogen bonds in the solid phase (double harmonic)—proof that benzocaine is pure.

The presence of the peak in the region 1700–1675 cm^−1^/1678.41 cm^−1^ is an indicator that signals the presence of the carbonyl group (C=O), which means that the ester structure of benzocaine was not altered during sample preparation; at the same time, this very intense band with low position denotes conjugation with the aromatic ring.

The peak is shifted to lower values (between 1670 and 1685 cm^−1^/1678.41 cm^−1^) as a result of the fact that the C=O group is directly related to the aromatic ring. The electrons delocalize, which weakens the C=O double bond and decreases its vibration frequency. A mesomeric effect of the amino group (-NH_2_) occurs, which sends electrons from the para position to the ring, further accentuating the decrease in the frequency of the carbonyl group (C=O). For a solid (crystalline) sample (the interactions between molecules), hydrogen bonds can pull the peak towards lower values, slightly to the right. The peak value of 1678.41 cm^−1^ confirms the presence of the aromatic ester group, so the sample has the correct benzocaine structure.

Correlating the peaks from 3418.35 cm^−1^/3337.81 cm^−1^ (amine), 1678.41 cm^−1^ (conjugated carbonyl), 1122.98 cm^−1^ (ester), and 844.65 cm^−1^/769.87 cm^−1^ (para substitution), the FTIR analysis spectrum corresponds to a high-purity benzocaine. The values are almost identical to those in the literature (e.g., SDBS or NIST database).

**Figure 8:**
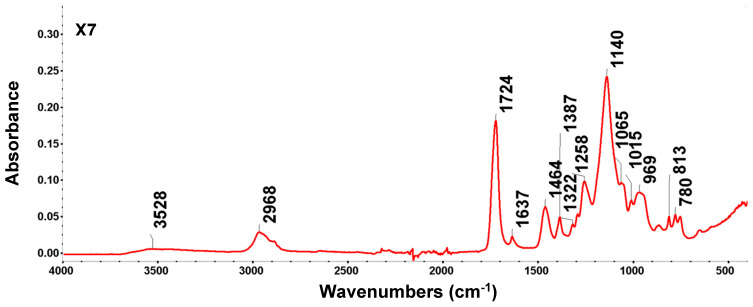
FTIR-ATR spectra of X7 resin.

X7 is a cross-linked, non-ionic aliphatic acrylic resin with a water holding capacity of 61–69%, an average area of 750 m^2^/g, and an average pore size of ~550 Å. Due to its aliphatic nature, it can adsorb non-polar compounds from aqueous systems and can also adsorb polar compounds from non-polar solvents. The particle size (430–690 μm, pore volume 0.5 mL/g) and specific surface area (380–500 m^2^/g, pore diameter 300–500 Å) of the resin influence the kinetics and operational capacity, and the pore diameter influences the degree of separation and recovery of the molecules. These characteristics of acrylic resin facilitate the desorption of bound molecules and reduce the amount of solvent required in the desorption stage. The FTIR spectrum reflects the polymeric structure of the acrylic ester type; it has intense bands specific to carbonyl groups and aliphatic chains, and the results obtained are presented in Figure 8 and Table 5.

In the region 1730–1630 cm^−1^/1723.52 cm^−1^ and 1637.27 cm^−1^, the peaks are very intense and confirm the acrylic nature of the resin. The value of 1723.52 cm^−1^ is the fingerprint of the carbonyl group (C=O) in the ester group.

The band at 3430 cm^−1^ is broad and slightly pronounced, indicating that the acrylic resin sample, X7, although hydrophilic, was thoroughly dried.

Absence of the aromatic ring: Unlike benzocaine, the X7 spectrum lacks the sharp bands above 3000 (aromatic C-H) and those at 1600 cm^−1^ (aromatic C=C), confirming its purely aliphatic nature. Changes in the intensity or position of these peaks (especially in the area of 1730–1630 cm^−1^) are used to monitor the efficiency of resin impregnation with various extractors (such as DEHPA or Cyanex).

Peak analysis confirmed the structure of cross-linked polymethyl methacrylate (PMMA), which is characteristic of the X7 matrix, but also indicated the presence of vibrations specific to the polymer backbone and, possibly, of adsorbed water.

FTIR analysis highlights the ester group and the acrylic backbone, which are the most characteristic features. Bands characteristic of the X7 matrix also appear in the aliphatic zone (CHx) where the peak (with a value of 2967.53 cm^−1^) is prominent for the asymmetric elongation vibration of the methyl and CH_2_ groups. Moreover, 1387.23 cm^−1^ is typical for the deformation vibration of the CH_3_ group. Another area of FTIR analysis is the fingerprint and the low-frequency region, where peaks occur at 1637.27 cm^−1^, specific to the deformation of the -O-H bond—a signal for the absorbed water. This denotes that X7 retains moisture very easily. A weak harmonic band (overtone) is indicated by the peak with a value of 1979.91 cm^−1^, often found in acrylic polymers without having major structural significance.

The peaks in the vibration region of the bands at 969.35 cm^−1^, 812.82 cm^−1^, and 779.83 cm^−1^ belong to the backbone vibrations of the polymer chain known in the literature as rocking or twisting modes of CH_2_ groups.

The spectrum depicts a clean acrylic matrix. The absence of new and dominant bands appearing in SDBS or NIST databases suggests a process of physical adsorption (by Van der Waals forces or hydrogen bonds) rather than a permanent chemical reaction that alters the structure of the polymer.

Therefore, the peaks at 1723.52 cm^−1^ (C=O) and 1139.94 cm^−1^ are still very clear and intense. This confirms that the polyacrylate backbone of X7 remained intact and was not chemically degraded during the adsorption process. The presence of water is indicated by the 1637.27 cm^−1^ peak—a classic indicator that the resin has been in contact with an aqueous solution. Even after a short period of drying, X7 retains water molecules in its macroreticular pores. The fact that no changes were observed, that no peaks appeared in the 1500–1600 cm^−1^ (specific to aromatic rings), and that there was no significant expansion of the band at 1258.06 cm^−1^ indicates that large organic molecules, such as phenols, dyes, or organophosphorus extraction agents, were not adsorbed. The peaks at 969.35 cm^−1^ and 779.83 cm^−1^ may be influenced by pore loading, but the fact that they are not broad or significantly shifted suggests that the adsorbed substance does not affect the skeletal vibrations of the polymer.

**Figure 9:**
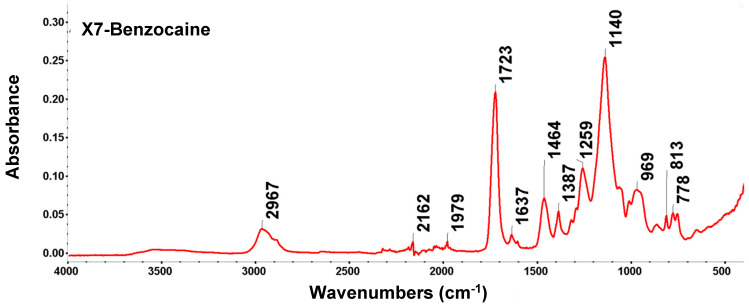
FTIR-ATR spectra of X7-Benzocaine.

FTIR spectrum analysis for the benzocaine-loaded X7 system reveals the interaction between the acrylic matrix and the adsorbate molecule. Since benzocaine is an ethyl ester of p-aminobenzoic acid, it brings specific functional groups (primary amine and aromatic ring) that overlap, or occur, near resin bands. The results obtained during FTIR analysis are presented in Figure 9 and Table 6.

In the pure resin, the band at 163.27 cm^−1^ corresponds to water adsorption. However, in benzocaine, the deformation vibration of the amino group (-NH_2_) and the vibration of the aromatic ring skeleton (C=C) occur. Their overlapping explains why this part is noticeable and well-defined after adsorption. The vibration regions at 812.82 cm^−1^ and 779.83 cm^−1^ are extremely important for benzocaine, corresponding to the out-of-plane deformation vibrations of the C-H bonds in a para-substituted aromatic ring (specific to benzocaine). Their presence suggest that the benzocaine is retained in the resin mass. The vibration region of the carbonyl bond elongation band C=O at 1723.52 cm^−1^ is composed of both the vibration of the acrylic ester, X7, and the benzoic ester, benzocaine, both of which have carbonyl groups. The fact that the tip is at 1723.52 cm^−1^ (slightly displaced from the standard PMMA value of 1730) suggests a disruption of the carbonyl groups, compatible with weak interactions between the amino group (-NH_2_) of benzocaine and the carbonyl group (C=O) of the resin. The peaks in the region of 1258.06 cm^−1^ correspond to the C-O stretching vibration in benzocaine and the resin ester, while the peak at 1139.94 cm^−1^ remains the most intense, being dominated by the polymer matrix, but its width may also indicate the presence of C-N bonds in benzocaine. The peak in the aliphatic region at 2967.53 cm^−1^ is attributed to both the methyl groups of the resin and the ethyl group (-CH_2_-CH_3_) in the benzocaine structure. The adsorption of benzocaine on X7 is mainly achieved by (i) dipole–dipole interactions between ester groups, (ii) hydrogen bonds between the amino group of benzocaine and the carbonyl oxygen of the resin, and (iii) Van der Waals forces between the aliphatic chains of the resin and the aromatic ring of benzocaine.

### 3.9. TG Analysis

**Figure 10:**
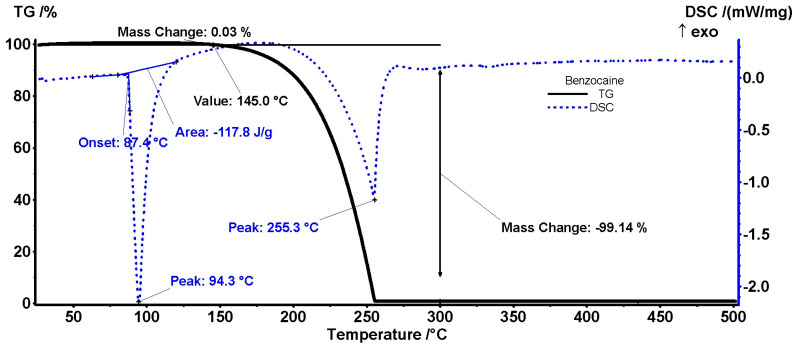
TG/DSC curve of benzocaine.

The process of benzocaine melting starts at 87.4 °C and ends at 94.3 °C. The measured melting enthalpy is 117.8 J/g, consistent with measurements in the literature data [43]. The sample is stable up to 145 °C (when the sublimation process starts), with the minimum associated endothermic effect starting at 255.3 °C and the process being complete at 256 °C (Figure 10).

The X7 can be considered stable up to 250 °C, with the recorded mass loss being 1.59%. Most probably, this process is caused by the elimination of residual humidity or by the oxidation of labile grafted moieties. Between 250 and 425 °C, the resin loses mass through multiple degradative–oxidative processes. The process starts with a small mass loss accompanied by a sharp and strong exothermic effect, with the maximum at 259.9 °C, indicating an oxidation reaction. The mass loss is continuous after that, with a net exothermic effect, indicating the overlapping of fragmentation and oxidation reactions.

**Figure 11:**
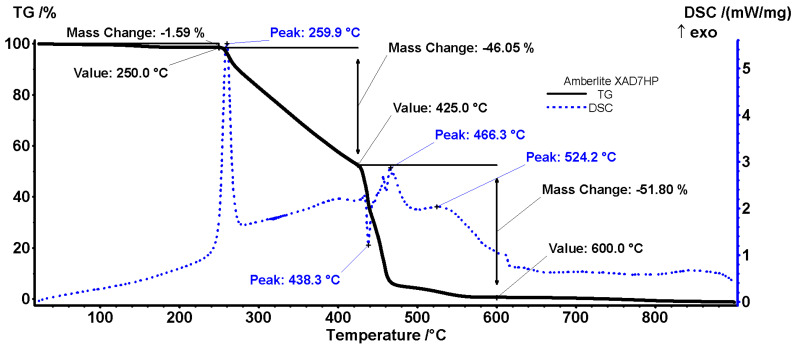
TG/DSC curve of X7.

The decomposition reactions become dominant after 425 °C, when a sharp endothermic effect is recorded at 438.3 °C. The mass loss continues with the oxidation of the resulting fragments, and then with the burning of residual carbonaceous mass. These oxidative processes are marked by exothermic effects at 466.3 and 524.2 °C (Figure 11).

**Figure 12:**
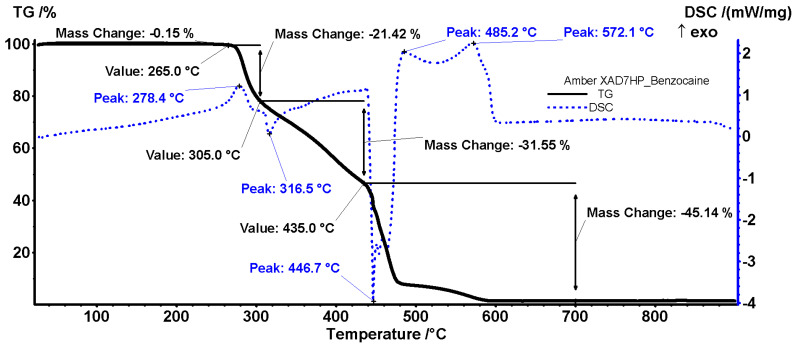
TG/DSC curve of X7-benzocaine.

The sample of X7 loaded with benzocaine exhibits a similar thermal pattern to the pristine resin. Nevertheless, some minor differences indicate the successful modification/retention of benzocaine (Figure 12).

Up to 265 °C, the sample exhibits no modification and is thermally stable. This contrasts with the pristine resin that loses 1.59% of its mass and can be considered stable only up to 250 °C. Most probably, the grafted moieties are binding the benzocaine molecule and therefore both become unavailable for oxidation. Binding the benzocaine also modifies the physical properties, inhibiting the sublimation.

Sample degradation starts at 265 °C, with a mass loss of 21.42%, and continues up to 306 °C. The process is accompanied by a small exothermic effect at 278.4 °C, indicating an oxidation reaction. After 305 °C, a weak endothermic effect at 316.5 °C (shown on the DSC curve) suggests a fragmentation reaction, followed by oxidative processes up to 435 °C. After 435 °C, the polymer chains are fragmented again, as indicated by the sharp and strong endothermic effect from 446.7 °C. The resulted fragments are oxidized thereafter, with the DSC curve indicating two exothermic effects at 485.2 and 572.1 °C.

## 4. Conclusions, Limitations, and Future Research

This study demonstrates that X7 is a highly efficient and resilient adsorbent for the removal of benzocaine from aqueous systems. The resin exhibited rapid uptake, high removal efficiency, and strong adsorption capacity across all operational conditions evaluated. Contact time, sorbent dosage, agitation speed, and initial concentration were identified as the primary parameters governing adsorption performance, whereas pH exhibit negligible influence, confirming that the process is dominated by hydrophobic and non-ionic interactions.

Equilibrium modeling results further supported these findings. The Langmuir isotherm provided the best fit to the experimental data (*R*^2^ = 0.9920), with an *R_L_* value indicative of favorable adsorption and high affinity. In contrast, the Freundlich model showed poor agreement (*R*^2^ = 0.5424), suggesting that it is not suitable for describing benzocaine adsorption on X7. The Temkin model indicated a decrease in adsorption heat with increasing surface coverage, while the Dubinin–Radushkevich model yielded an adsorption energy of 3.54 kJ/mol, consistent with a physical adsorption mechanism. Overall, the Langmuir model best describes the system, suggesting spontaneous adsorption on a homogeneous X7 surface.

Desorption experiments revealed that acidic and ethanol-based media significantly enhanced benzocaine release, whereas alkaline conditions were less effective. The HCl–ethanol mixture achieved the highest desorption efficiency (97%), demonstrating that X7 was regenerated in a single adsorption–desorption cycle. However, long-term reusability cannot be inferred from a single cycle and requires further investigation.

Material characterization supported the adsorption behavior observed experimentally. FTIR-ATR confirmed the presence of benzocaine on the resin surface without evidence of chemical modification, while TG/DSC analysis verified the thermal stability of the resin and the incorporation of benzocaine within its porous structure.

Despite its strong performance, several limitations must be acknowledged. This study was conducted using single-solute systems, whereas real wastewater contains complex mixtures of pharmaceuticals, organic matter, and competing ions that may influence adsorption behavior. Experiments were performed under controlled laboratory conditions, leaving matrix effects such as turbidity, ionic strength, and dissolved organic carbon unassessed. Additionally, adsorption experiments were carried out at a single temperature (25 ± 2 °C), preventing the calculation of thermodynamic parameters.

To advance the practical applicability of X7 in wastewater treatment, future research will focus on (i) evaluating adsorption performance in real or simulated wastewater matrices containing multiple pharmaceuticals and competing organic compounds; (ii) assessing the feasibility of integrating X7 into continuous-flow systems, fixed-bed columns, or hybrid treatment technologies; (iii) conducting techno-economic and environmental assessments to determine scalability and sustainability for industrial or municipal applications; (iv) performing multi-cycle adsorption–desorption experiments to evaluate operational stability and long-term reusability; and (v) conducting adsorption studies at multiple temperatures to enable a comprehensive thermodynamic characterization of benzocaine adsorption on X7.

## Figures and Tables

**Table 1:** Experimental parameters of adsorption isotherm.

**Langmuir**		**Temkin–Pyzhev**	
*Q*_0_ (mg/g)	147	*A* (L/mg)	88.4
*b* (L/mg)	5.2	*b_T_* (J/mol)	112
*R_L_*	0.0003	*B*	26.7
*R* ^2^	0.992	*R* ^2^	0.741
**Freundlich**		**Dubinin–Radushkevich**	
*K_f_* (mg/g)	116	*q_m_* (mg/g)	183
1/*n*	0.36	*β* (mol^2^/kJ^2^)	3.98 × 10^−8^
*n*	2.75	*E* (kJ/mol)	3.54
*R* ^2^	0.5424	*R* ^2^	0.8475

**Table 2:** Kinetics parameters of benzocaine adsorption onto X7 resin mass.

**PFO**	$k_{1}$ (min^−1^)	0.060
	$q_{e,\mathrm{calc}}$ (mg/g)	98.7
	$R^{2}$	0.9550
**PSO**	$k_{2}$ (g/mg·min)	0.001
	$q_{e,\mathrm{calc}}$ (mg/g)	100.4
	$R^{2}$	0.9990

Where *k*_1_: PFO rate constant, associated with diffusion-controlled or physisorption processes; *k*_2_: PSO rate constant, associated with chemisorption or strong surface interactions; *q*_*e*,*calc*_: theoretical equilibrium adsorption capacity of X7 predicted by each model. *R*^2^: correlation coefficient of kinetics model; higher values confirm the most appropriate kinetic model.

**Table 3:** Comparative adsorption performance of pharmaceuticals on polymeric materials and different adsorbent materials.

Pharmaceutical	Adsorbent	Adsorption Capacities (mg/g)	Experimental Conditions	References
Sulfamethazine (SMZ)	Purolite C100E (strong acid cation-exchange resin)	338.92 ± 21.46 (Langmuir model)	pH 5; resin dosage 1 g/L; contact time 150 min; initial SMZ concentration 20–280 mg/L.	[39]
Tetracycline hydrochloride (TCH)	PCC-NH_2_-βCD-MOF (polyethylene glycol and citric acid cross-linked amino-β-cyclodextrin MOF)	278.2 mg/g (experimental) 526.3 mg/g (Langmuir theoretical at 25 °C)	Optimized pH; porous, negatively charged surface; temperature-dependent kinetics; contact conditions optimized; spontaneous and exothermic (ΔH° = −34.85 kJ/mol); 78% capacity retained after four cycles; 82% removal in simulated wastewater.	[40]
Bisphenol A (BPA)	tris(4-formylphenyl) amine (TFPA) onto β-cyclodextrin (β-CD) to synthesize a β-cyclodextrin polymer (TFPA-N-β-CD).	164.73	pH not specified (single-component system); adsorption follows pseudo-second-order kinetics and Sips isotherm; dominant mechanism: host–guest inclusion via β-CD cavity.	[41]
Methylene blue (MB)		272.03		
Tetracycline (TC)		33.89		
Sodium diclofen3ac	Activated carbon from PET (H_3_PO_4_-activated)	34.06 mg·g^−1^ (experimental) 200 mg/g (model-estimated optimum)	Activation with H_3_PO_4_, ratio 2:1 (H_3_PO_4_: carbon); specific surface area 542.97 m^2^·g^−1^; pores 15.3–120 nm (meso–macro); optimal conditions: 25 °C, 25 min; 4 kinetic models and 2 isotherms tested; maximum capacity estimated by kinetic/isothermal simulations.	[42]
Trimethoprim	X7	Not reported as mg/g; 70% removal in 2 h	Adsorbent dose 1–3 g/L; stirring 80–240 rpm; pH 2–9 (pH strongly affects removal); 20–60 °C; 25–75 mg/L; best-fit model: pseudo-second-order.	[27]
Carbamazepine		Not reported as mg/g; 85% removal in 2 h		
Naproxen		Not reported as mg/g; 60% removal in 2 h		
Carbamazepine	X7 (acrylic ester resin)	97 mg/g	pH 7; Langmuir/Freundlich/D–R models; E = 8.3–10.1 kJ mol^−1^; acidic drug—adsorption decreases at higher pH; capacity decreases in mixtures.	[36]
Trimethoprim		54 mg/g		
Ketoprofen		45 mg/g		
Naproxen		43 mg/g		
Benzocaine	X7	≈140 mg/g at equilibrium (60 min)	Contact time 0–90 min (equilibrium at 60 min); dosage 0.01–0.05 g; agitation 25–200 rpm; pH 5–9; initial concentration 100–700 mg/L; removal efficiency up to 99.9%; adsorption mechanism predominantly hydrophobic and non-ionic; best-fit isotherm: Langmuir (*Q*_0_ = 147 mg/g, *R*^2^ = 0.992); kinetics: rapid external mass transfer, desorption highest in HCl–ethanol (97%).	This study

**Table 4:** Summary of FTIR bands and functional group vibrations in benzocaine.

Functional Grouping	Vibration Mode	Position (cm^−1^)	Observations
Amino groups (-NH_2_)	Asymmetrical N-H bond stretch band and symmetrical N-H bond tension	3420–3220/3418.35; 3337.81; 3220.25	Two distinct and sharp peaks characteristic of the primary amine group (R-NH_2_) appear. The appearance of the peak at 3220.25 cm^−1^ is the result of intermolecular hydrogen bonds in the solid phase (overtone proof that benzocaine is pure).
Alkyl groups (-CH_3_)	Asymmetrical stretch band (-CH_3_) and symmetrical (-CH_2_-)	2980–2900/ 2983.64–2899.81	Aliphatic stretching of the ethyl-CH_2_-CH_3_ group in the remaining alcohol.
Carbonil (C=O)	Stretch tape	1700–1675/1678.41	A very intense band with a low position due to the conjugation with the aromatic ring; the peak corresponds to the carbonyl group.
Aromatic (C=O)	Aromatic ring stretch strips	1600; 1580; 1440/1594.39; 1574.01; 1440.97	Specific bands of the substituted benzene ring.
Ester (C-O-C) and C-N	Stretch bands of the bond between the aromatic ring and the amine nitrogen	1100–1170/1108.55; 1122.98; 1169.73	Intense band specific to the simple bond between carbon and oxygen within the ester; Ester Grouping Specific Band.
Ester (C-O-C)	Stretch band	1270–1150/1271– 1169.73	Intense band corresponding to the C-O bond in the ester structure.
Amino (-NH_2_)	Scissoring tape	~1600/1594.39	Indicates an overlap of aroma ring vibrations.
Aromatic (C-H)	Out-of-plane deformation band	850–750/844.65; 769.87	Indicates substitution in the para (1,4-disubstituted) position. The presence of this part confirms that the aromatic ring has two groups attached opposite each other (nitrogen and carbonyl), according to the structure of benzocaine.

**Table 5:** Summary of FTIR bands and functional group vibrations of X7 resin.

Functional Groups	Vibration Mode	Position (cm^−1^)	Observations
Hydroxyl groups (-OH)	stretching band	3700–3100	Maximum around 3430 cm^−1^; a wide band attributed to the elongation vibrations of the hydroxyl (-OH) groups coming from the adsorbed water, reflecting the hydrophilic nature of the resin.
Carbonyl (C=O)	stretch band	1730–1630/1723.52; 1637.27	Very intense and sharp tape, specific to the acrylic ester; C=O bond elongation tape in the acrylic ester structure.
Aliphatic (CH, CH_2_, CH_3_)	stretching bands	2975, 2930, 2890/2967.53	Three distinct bands corresponding to the asymmetrical and symmetrical vibrations of the aliphatic group bonds (C-H, methyl and methylene).
Adsorbed water (-OH)	stretch band	~3430/	A broad band due to moisture retained in the macroporous structure.
Deformation (CH_3_)	stretching bands	1477;1390/1463.96; 1387.23	Medium bands generated by methyl (-CH_3_) groups on the polymer backbone; C-H bond strain bands, with the peak of 1387.23 being typical for methyl (-CH_3_) grouping.
Ester (C-O-C)	stretch band	1260–1150/1258.06; 1139.94	Intense bands associated with the C-O bond in the basic structure; the vibration of elongation of the ester bond (C-O-C); the very intense peak is characteristic of the elongation vibration of the C-O bond or the C-C-O couplings in the matrix-specific cross-linked polymethyl methacrylate (PMMA) structure.

**Table 6:** Summary of FTIR bands and functional group vibrations of benzocaine-loaded X7 HP.

Peak Observed (cm^−1^)	Structural Attribution	Peak Displacement
2967.53	Aliphatic CH elongation	The slight shift to the right comes from both the resin and the ethyl benzocaine group.
1723.52	Carbonyl (C=O) ester	Pure benzocaine exhibits C=O at 1680 cm^−1^. The value of 1723.52 cm^−1^ shows an overlap with the resin ester (1745 cm^−1^), indicating interactions by hydrogen bonds.
1637.27	NH_2_/C=C deformation	Area characteristic of benzocaine (aromatic ring and amine group); in pure resin, this area is often occupied only by adsorbed water.
1463.96	Deformation CH_3_	Displaced from 1477 cm^−1^ (pure resin), confirming the change in the chemical environment of the matrix.
1139.94	Elongation C-O	Very intense section, typical for the acrylic skeleton of the X7 HP.
812.82/779.83	Aromatic ring	Clear adsorption of approval; these are the out-of-plane deformation vibrations for the para-substituted benzene ring of benzocaine.

## Data Availability

The original contributions presented in this study are included in the article. Further inquiries can be directed to the corresponding author.
